# “You’re actually part of the team”: a qualitative study of a novel transitional role from medical student to doctor

**DOI:** 10.1186/s12909-023-04084-9

**Published:** 2023-02-15

**Authors:** Edmiston Natalie, Hu Wendy, Tobin Stephen, Bailey Jannine, Joyce Caroline, Reed Krista, Mogensen Lise

**Affiliations:** 1 School of Medicine, Western Sydney University, Sydney, Australia; 2 University Centre for Rural Health, Lismore, Australia

**Keywords:** Medical student, Curriculum, COVID-19, Transitions, Teamwork, Experiential learning, Assistantships, Clerkships, Electronic medical record, Qualitative analysis, Activity theory, Assistants in Medicine

## Abstract

**Background:**

Optimizing transitions from final year of medical school and into first post graduate year has important implications for students, patients and the health care system. Student experiences during novel transitional roles can provide insights into potential opportunities for final year curricula. We explored the experiences of medical students in a novel transitional role and their ability to continue learning whilst working as part of a medical team.

**Methods:**

Novel transitional role for final year medical students were created in partnership by medical schools and state health departments in 2020 in response to the COVID-19 pandemic and the need for a medical surge workforce. Final year medical students from an undergraduate entry medical school were employed as Assistants in Medicine (AiMs) in urban and regional hospitals. A qualitative study with semi-structured interviews at two time points was used to obtain experiences of the role from 26 AiMs. Transcripts were analyzed using deductive thematic analysis with Activity theory as a conceptual lens.

**Results:**

This unique role was defined by the objective of supporting the hospital team. Experiential learning opportunities in patient management were optimized when AiMs had opportunities to contribute meaningfully. Team structure and access to the key instrument, the electronic medical record, enabled participants to contribute meaningfully, whilst contractual arrangements and payments formalized the obligations to contribute.

**Conclusions:**

The experiential nature of the role was facilitated by organizational factors. Structuring teams to involve a dedicated medical assistant position with specific duties and access to the electronic medical record sufficient to complete duties are key to successful transitional roles. Both should be considered when designing transitional roles as placements for final year medical students.

**Supplementary Information:**

The online version contains supplementary material available at 10.1186/s12909-023-04084-9.

## Background

New doctors face numerous challenges during transition from student to professional practice [1, 2]. Concerns relate to a feeling of responsibility, fear of being incompetent and fear of making mistakes [3]. While it has been acknowledged that a period of transition is inevitable [4], optimisation of this transition has important implications both for the doctor undertaking this transition and the system in which they work [1, 5, 6]. The final year of medical school both synthesises medical knowledge and prepares students to work as post graduate year one (PGY1s) doctors where they will manage patients under supervision.

Preparedness, or the feeling of confidence at the beginning of PGY1, has been found to be higher for students who have had more real life experiences [7]. The development of a professional identity as a doctor is also facilitated by more time in hospitals and with patients [8]*.* Importantly, competence and expertise are developed through practice. Evaluation of medical students has shown students show classic developmental progression towards competence [9]. Students are most challenged by the role of health care manager [9] but competence in this role increases sharply over the transition period from student to doctor [10]. Patient management requires adaptive expertise which is ultimately learnt through supervised involvement in this work.

Many countries have re-positioned final year medical students as part of the medical care team to facilitate experiential learning [11]. In the United Kingdom, assistantships are final year medical students who work within a team in an unpaid capacity [12]. In the Netherlands, “semi-physicians” rotate through relatively long terms during the final transitional year of medical school and perform similar tasks as PGY1s [13]. In Australia as well as the United States, longitudinal integrated clerkships place students alongside doctors to be authentic participants in patient care [14]. Potential benefits of experiential learning include better preparing PGY1s to be able to work with increased professionalism and expertise, particularly adaptive expertise [15].

In 2020, the COVID-19 pandemic disrupted established clinical placements as restrictions were placed on non-essential staff attending health facilities [16]. At the same time the need for health care providers had never been so high, so medical students were considered to be a potential surge workforce from early 2020 [17]. Novel transitional roles have been developed worldwide to enable final year medical students to join the medical workforce prior to formal completion of their medical degree [18, 19]. Student experiences during these roles can provide insights into opportunities for final year medical school curricula to optimize the transition to PGY1. For this study, we explored the experiences of medical students in a novel transitional role and their ability to continue learning whilst working as a paid member of a medical team. The results of this study will contribute to the knowledge base about extracting maximal benefit from clinical placements and experiential learning.

## Methods

The research methodology was qualitative research using individual and group interviews. We report our study using COREQ reporting guideline [20].

### Study context

In Australia successful completion of a medical degree is followed by PGY1 [21], prior to full registration as a medical practitioner.

In New South Wales (NSW), the most populous state of Australia, a new Assistant in Medicine (AiM) role was created in partnership between medical schools and health departments. In this official role, students were employed for up to 32 h per week with a salary equivalent to 75% of the PGY1 rate. For the remaining day of each week, AiMs participated in medical student teaching. The responsibilities of the AiMs could vary but AiMs were not able to prescribe medication. The AiM role was rapidly developed and introduced in less than three months.

In 2020, medical students from a direct entry five year medical program in NSW, were offered the opportunity to work as AiMs in place of their usual final year rotations, which consisted of a 35 h study week with clinical placements [22], including rotations in Medicine, Surgery, General Practice, Indigenous Health and Critical Care. AiMs were allocated positions at a variety of metropolitan hospitals and at two rural regional hospitals. One of these regional hospitals, (Region A) usually employed PGY1 doctors, whereas the other regional hospital (Region B) did not. AiMs placements varied in length from 12 to 24 weeks, which are longer terms than the typical student rotations of 5–9 weeks. Typically, the AiMs did two placements with two different clinical teams. The AiM role was offered to volunteering students and contributed toward completion of the medical course [23]. Only final year students who had passed all assessment requirements including final year progression points, with no professionalism concerns were eligible to apply for AiM positions. Prior to commencement AiMs undertook an intensive week of skill development. AiMs continued with educational activities, especially workplace based assessments and engaged with university learning platforms. A total of 83 AiMs were recruited; 60 were placed in metropolitan hospitals; 6 in Region A and 17 in Region B.

### Research team

The research team consisted of the listed authors who were all university academics at the time of the study. All but one (ST) are female. The team comprised experienced qualitative researchers working in medical education research (LM, JB, WH, CJ) and clinical researchers involved in medical education (ST, NE). The researchers were known to the participants through their positions as medical or research educators in most cases, however none of the researchers were directly supervising or grading participants at the time of the study.

### Study design

Our study was nested within a larger realist evaluation of the AiMs program [24] and aimed to provide detailed contextual findings across three sites and two time points. The first interview was designed to occur mid-way through the initial AiM placement. The interview explored student self-reported experiences in the early weeks of the AiM role, including expectations, preparedness, confidence, motivations and sense of professional identity development, opportunities to undertake tasks, and learning activities. Training support, self-care and wellbeing were also explored. See Additional file 1: Appendix A – Round one interview questions.

Second interviews focused on student self-reported experiences in the later part of the AiM role, including expectations, confidence and sense of professional identity development, opportunities to undertake more advanced tasks and learning activities. Their experiences of assessments of knowledge and skills were also explored, along with ongoing training support, self-care and wellbeing. See Additional file 1: Appendix B – Round two interview questions.

Interviews were either group or individual and were conducted via video conference and audio -recorded. The interviews were conducted by LM, JB, KR, WH, NE and CJ either individually or in pairs. ST did not interview due to his role as one of the architects of the AiM program. The duration of each interview was 45 to 60 min. Audio recordings were transcribed and de-identified.

### Participants and recruitment

All 83 students undertaking an AiM role were invited to participate in this study and there were no exclusion criteria. An interview was offered to AiMs within a few weeks of commencement in the role. Recruitment was by notifications on the student online learning platform and by verbal invitation. Verbal invitations occurred when students attended a weekly university educational session. Participants in this first interview were asked for their consent to be invited directly via their student email to a second interview.

Informed consent was obtained prior to data collection. This study was approved by Western Sydney University Human Ethics Committee ID No. H9989.

### Data analysis

Deductive thematic analysis was utilized in this study, following the thematic approach as described by Braun and Clarke [25]. Prior to application of the deductive thematic approach, team members familiarized themselves with the data and met to discuss initial impressions. The de-identified transcripts underwent initial coding by NE to identify experiences of the AiM role and the responsibilities and opportunities attached to the role. This initial familiarization and exploration used an inductive approach, to allow exploration of lines of inquiry and theory as described in the realist protocol [24]. The research team met again and discussed the themes identified, before progressing to a deductive approach using Activity theory.

Activity theory was used as a sensitizing lens for exploring system level issues and positioning AiMs within a larger system. Activity theory is an approach utilized in developmental research [26], that has previously been applied to both medical education [27] and to newly graduated doctors [28]. An Activity system is a system working together to produce an object. Objects can be considered as the true motivation of the system, generally long term and durable in nature, and systems can be differentiated by their objects or motives [26]. Subjects are actors or participants in the system who are motivated or aiming to achieve the object of the system [29]. Activity theory is useful in revealing how both the properties of tools and culture impact on the ability to achieve the object of the system. Whilst subjects are motivated towards the object, the ability to achieve it is mediated, or impacted by the tools, rules and division of labour that apply to the system. It is through this mediation that an outcome is produced, that differs from the object. Objects, although long term in nature, are not fixed but can change over time and are often contested [26]. The constant change within an activity system produces contradictions or tensions within the system, but these provide opportunity for rich learning [27]. In addition, each of these elements interact with each other, and these interactions can be complementary or contradictory.

A deductive thematic analysis using Activity theory was implemented, with NE conducting second round of coding, and defining and naming themes. Researchers met again to discuss the Activity theory themes and develop the final themes. Analysis was conducted utilizing NVivo, Version 12 [30].

### Reflexivity

The researchers acknowledge their position of authority within the medical school structure. The interviewers were known as members of the medical school to the participants. The interview schedule emphasized the novel nature of the role and the enquiring nature of the interviewers into this new program. The participants were acknowledged for their opportunity to educate the research team about a program that was new to both participants and researchers.

The data analysis was led by NE, who had commenced employment with the medical school within the same year that the interviews took place. This allowed for some objectivity towards the analysis. Multiple meetings with more experienced members of the research team allowed for in-depth understanding of context. Activity theory was discussed at length with WH, an experienced qualitative researcher in medical education.

## Results

Initial data collection took place in August and September 2020 via a total of five group interviews. The second round of data collection took place in November 2020, consisted of both individual and group interviews (Table 1).

**Table 1:** Participants

	Round 1 *N* = 29 Participants (P)	Round 2 *N* = 22 Participants (P)
Region B	1 Group interview	1 Group interview
	P1-17; 7 Male, 10 Female	P1-17; 7 Male, 10 Female
Metropolitan	3 Group interviews	2 interviews
	P18,19; 2 Female	P18; 1Female
	P20-22; 2 Female, 1 Male	P19; 1 Female
	P27-29; 3 Female	1 Group interview
		P21, 28; 1 Female, 1 Male
Region A	1 Group interview	1 interview
	P23-26; 2 Female, 2 Male	P25; 1 Male

**Fig. 1:**
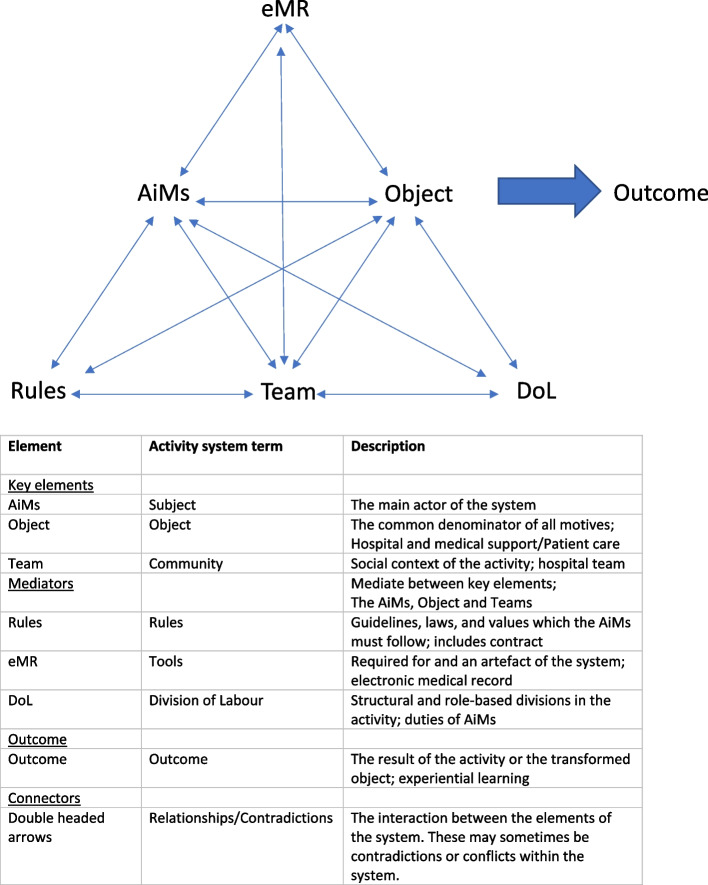
Activity System for AiMs (Adapted from Engestrom (1))

The object of the AiM system (Fig. 1) was to support the hospital and medical systems during the COVID-19 pandemic and to provide patient care, whilst the outcome of the system for the purposes of this analysis is the experiential learning of the AiMs. The AiMs worked within the existing hospital community and the relationship between the AiMs, the community of practitioners and their work was mediated by three factors; tools, rules and the division of labour. This lens revealed the unique role of AiMs, the mediating role of the eMR as the main tool and payment and contractual arrangements as a key rule.

### *The subject’s unique hybrid role was defined by the object and supported by the community*

In volunteering for an AiMs role, the students were aware of their ability to support a system struggling with COVID disruptions. The object of the AiMs system varied throughout the program. In early interviews AiMs strongly identified an objective of supporting a team or hospital system; acting as “helper” within the team, to provide support and free up PGY1s to do more crucial work. AiMs were willing to be responsible for tasks such as documentation and requesting consults, which they viewed as administrative rather than medical, as they felt that this was important to help the team as a whole.

*It's really to support the teams there, especially the (PGY1s) who are, you know, spread thin… Yeah, and just sharing the workload.*

Metropolitan, Round 1, P4

By the second interview AiMs were more confident with the administrative tasks and were able to see how they contributed as part of a team to provide patient care. AiMs identified the objective to care for patients, and their impact on patient outcomes. Increasingly they developed a professional identity as doctors who cared for patients.

The AiMs viewed their role as a unique hybrid role. They consistently presented themselves as *different from medical students,* referring to themselves as AiMs, rather than as students. Students were positioned as learners whereas AiMs were identified as doing clinical tasks whilst still learning. The role of an AiM was frequently articulated as someone who “did” rather than someone who just “learnt”. By listing the clinical activities that they did, AiMs were clearly identifying themselves as people who “did” things rather than as people who learnt how to do things.

*In the emergency department, we are seeing our own patients, essentially, like working them up, history, examination, doing initial investigations….. conduct the procedural skills as well that are within our scope.*

Region B, Round 1, P1

AiMs could not legally prescribe: the inability to “do” tasks such as prescribing for the patient was a clear indication that AiMs were not yet doctors. AiMs experienced some frustration at not meeting the expectations, of themselves and others, that AiMs should be able to “do”. When unable to do tasks AiMs were likely to revert to the student role, considered an inferior position. AiMs noted that medical students were usually given tasks for learning, whereas the tasks delegated to the AiMs were ones that needed to be done for patient care. AiMs were proud of their contribution and developing professional identity.

The unique role was frequently acknowledged from the viewpoint of other team members. AiMs were usually referred to as “doctors” by nursing staff or patients. The medical team considered AiMs as close to PGY1s. AiMs recognized that they were trusted; enabling them to do tasks semi-independently. Trustworthiness was embedded in the role of the AiM but was reinforced through the duration of the rotations as the AiMs demonstrated their ability to perform the tasks. Despite some expressions of ambivalence about their responsibilities, AiMs generally felt confident to do the tasks within the scope of their role. Yet, the hybrid nature of the role, gave the opportunity to still be considered as a learner when they were uncertain about a task. They also felt that other people would be supervising their work and in this way they would be protected from making mistakes. This gave AiMs confidence because the unique role did not have the full responsibilities of a PGY1.

### *The electronic medical record is the fundamental tool for junior doctors and access is crucial for successful AiMs*

Many of the activities of junior doctors such as documenting ward rounds, ordering consults and preparing discharge summaries, take place within an electronic medical record (eMR). Permission to use this eMR varied with location. Access to the program and a level of delegation similar to a doctor was seen as very important to the AiMs’ ability to contribute to the team.

*Now we’re able to actually write and sign off on notes and discharge summaries as physicians, rather than ….. as medical students and that’s been extremely helpful because ….. we can do a lot of clinical documentation, which is a key part of a (PGY1s) role.*

Region A, Round 1, P23

In contrast, the lack of access to this system for some AiMs impeded the ability to contribute. Lack of access was particularly problematic in Region B although some metropolitan locations also experienced lack of access, particularly at the start of the AiM program.

*our eMR access, which never got fixed. Which slowed the whole team down a lot …., we didn't have growth charts, which is the most ridiculous thing because it's really fundamental to paediatrics. And I don't see how us or medical students having growth charts is going to harm children in any way.*

Region B, Round 2, P6

Activity theory acknowledges that access to the key instrument is crucial to the success of the entire system. For the paediatric AiM system, the lack of access to the growth charts within eMR, seen as a fundamental aspect of paediatric medicine, caused inefficiencies and was seen as “ridiculous”. In addition to practicalities, access at a level similar to a junior doctor was a symbol of the AiMs role as part of the medical team, particularly identifying them to other doctors. The dissatisfaction with lack of access reflects not only the inefficiency of the system but a lack of recognition. It impeded true integration within the team and the opportunity to learn the crucial skill of operating the eMR. Where access was allowed, the absence of co-signatures as would be required for students made the AiM responsible for the patient care via eMR and motivated them to really understand the patient’s care.

### ***The organisational rules for AiMs including contracts, job description, expectations to work, and payment all enabled AiM roles***

Being paid was a prominent theme in the first interviews, as it was their first professionally paid position within medicine. Payment obligated contribution to the team and enabled delegation of tasks not merely for educational value. Payment acted as a facilitator for the unique role both as an obligation to the AiM and a motivator for the team.

*we're getting paid. I think those expectations from on the part of the teams and the hospital staff and also myself that you know you have to work professionally, because it was a job*

Metropolitan, Round 2, P21

Payment was an important symbolic factor defining the uniqueness of the AiMs role. Some AiMs indicated that junior doctors voiced to the AiMS and to other medical staff, that they themselves had not had payment for being a final year medical student. Possibly expressing a degree of jealousy, these statements reinforced the obligations of the role including an obligation to be present at the hospital for prescribed hours and to do tasks regardless of the learning opportunities afforded by them. The junior doctors, perhaps unintentionally, were supporting the AiM role by encouraging the team to view the AiMs as different from students.

Whilst payment prioritised clinical work, the work actually enriched learning opportunities. As a result of the expectation to work, AiMs were “acting up”, being expected to do clinical tasks. The resultant increased exposure to the clinical environment, both in terms of duration and centrality, provided an opportunity to gain confidence as a future doctor.

*now that you’re on the payroll, you are actually a part of the team. And so suddenly it becomes more appropriate for them to be allocating tasks for them to up skill you, and mentor you so that you perform those tasks to their required standard…. And because of that, because of the exposure that you're getting, because you're reinforcing those skills on a much more frequent basis, that's actually leading to increased confidence overall.*

Region B, Round 2, P12

Informal rules, such as social reciprocity and responsibility, became more obvious to AiMs as the terms progressed. They recognized that it was a privilege to be in the role and that it was a unique opportunity. They felt that doing a good job as an AiM was important so that AiM type roles might be considered for future years, particularly important to Region B AiMs.

### *The division of labour within the medical team was key to successful AiM roles*

AiMs identified that some positions did not have sufficient tasks for an AiM, whilst successful positions had ample tasks for juniors. The teams quickly recognized what tasks could be completed by AiMs and delegated these, leaving the junior doctors free for tasks such as prescribing that AiMs could not do. Some AiMs often swapped between teams in order to provide the most efficient use of their labour. In return their contribution was valued with teams voicing their appreciation.

*the RMOs and the registrar say that oh yeah he's, you know, able to do this, this and this. So then we will delegate some of these responsibilities to him, and we're happy for him to, you know, do all of those jobs and then hand over back to us*

Region A, Round 1, P25

Team structures including junior doctors not only allowed for efficient division of labour, it also meant near peers were present. AiMs were able to become team members and were quickly able to function effectively and learn the role. Conversely the absence of a near peer made the AiM role challenging as the gap between seniors and AiMs was seen as too large.

*It's nice having (a PGY1) and having a resident or someone who's a (junior) on both teams because I feel like I can always go to them ….. They're kind of a middle ground between me and the higher-ups on the team.*

Metropolitan, Round 1, P23

### *AiMs learning as an outcome was enriched despite some challenges*

Whilst medical students were positioned as learners, AiMs primary role was to fulfil their work obligations. Therefore, initial priorities of the AiMs were to learn to do the necessary tasks with proficiency; tasks which were closely aligned with the skills that would be useful as a PGY1 in future. For many AiMs, the opportunity to learn the PGY1 role was a motivating factor in volunteering for the program, as PGY1 was perceived as stressful and even “scary” and being an AiM would make this transition less daunting. Yet, AiMs identified that they still had obligations to complete their medical education through catching up on placements in spare time, studying theory and completing documentation about competencies on university systems. Despite time challenges, they recognized the importance of consolidating their medical knowledge in their final year of study.

*two types of learnings and both are equally important. I feel like I want to be able to perform as a good (PGY1) next year, but then also be a good doctor.*

Metropolitan, Round 2, P28

For the most part, the learning environment of the AiMs was considered to enrich both theoretical and PGY1 role learning. After a pre-commencement intensive week, AiMs did the vast majority of their learning within the work environment. Junior doctors were frequently cited as people who taught them how to do their tasks. In return for teaching the AiMs, the junior doctors were able to share their workload with the AiMs.

Consultants and registrars were generally a source of support and ensured that AiMs felt part of the team but were noted to be unfamiliar with the systems that the AiMs and junior doctors utilized. In addition to demonstrations, AiMs received real time feedback and mentoring from their team, to ensure they were able to contribute to the team and provide safe care of the patients. AiMs recognized that PGY1s need to be professional and have management skills to manage their own workload. They recognized the benefits of same in their own role. Managing patients and being a part of the team providing care was seen as a crucial immersive activity to build confidence in the skills they would require as junior doctors.

*builds confidence knowing you’re learning how to be on the ward by yourself, especially in surgical term where your (registrars) aren’t that available, like feeling confident, just making decisions yourself. Time management; managing like a high stress load*

Region B, Round 2, P5

Learning was enhanced by the longer placement duration, which enabled the AiMs to understand the complete patient journey. They experienced the important roles of other health professionals in the care of patients. By staying the full work day, AiMs were frequently present when patients deteriorated and acute care was required in the late afternoon. In addition, AiMs were given opportunities to present cases, for example at morbidity and mortality meetings and this required them to extend their theoretical knowledge.

In summary, the role of AiMs was truly a unique novel role that facilitated experiential learning, and maintained security of the student role, whilst contributing to the workforce.

*A little bit of a hybrid, I would say I think I've still got the privileges of being a medical student …. But at the same time, I feel like I can step up and do some of the roles that doctors can do as well.*

Region A, Round 2, P25

## Discussion

This study has provided insights on the novel transitional role of AiMs and its impact at a crucial stage in medical student learning. Activity theory highlights that access to the eMR and a team structure that allowed division of labour facilitated the experience.

The success of the role is supported by the state health department’s evaluation of the role [23] and the prompt reinstitution of the role in the 2021 Covid-19 Delta wave. This evaluation contained multiple viewpoints other than the AiMs and found that the AiMs functioned like PGY1s within a few weeks and were considered valuable members of the team [23]. A strength of our study is that it looks at two time points in the journey of AiMs to document the developmental journey of students in this penultimate stage towards graduation.

Transitioning from student to doctor is a process that occurs over time with the steepest transition at the start of PGY1. Feeling prepared to manage patient care has been found to be the aspect of preparing for working as a doctor that takes the most time to develop [9, 10]. Given the complexity of the task and the need to be adaptable, it is appropriate that final year students be exposed to as much of this task as possible. Management includes medical documentation within the eMR, a key competency for new doctors particularly from the view point of others in the care team such as nurses [31].

Access to the eMR was determined on a local level and could vary from the same as a medical student with all documentation requiring co-signatures to a higher level that allowed documentation and some ordering of investigations without a countersignature. Almost half of the Directors of AiMs, responsible for implementing the AiM program in hospitals, reported AiMs had medical student level of access and only 11% allowed discharge summaries to be completed without countersignature [23]. Our study showed that AiMs with access to eMR discharge summaries, were subsequently allocated and trained to do these tasks. Other authors have found that access to the prescribing “tool” can be successfully implemented for final year students [2]. Access to the tool facilitates learning of the task, enabling learning of the skills necessary for junior doctors.

Evaluation of similar programs have concluded that the quality of the teamwork experience [12] facilitated the success of assistantship roles. Team structures that supported trust development were particular efficacious for AiMs. The decision to entrust an activity to a student is affected by the quality of the relationship between learner and supervisor, both in duration and degree of reciprocal trust. Longer attachments allow for more trust to develop [32] but for the AiMs the obligations and structure of the role meant that they typically earned trust sooner than might occur as a student. Once entrusted, AiMs could be actively involved in managing patients. For medical students, a virtuous cycle exists whereby students that are the most capable are perceived as doctors and hence more integrated into the team and are able to further develop skills [9]. For AiMs, the perception as hybrid team members; not doctors but clearly not students, slotted them into this virtuous cycle at an advanced level.

It is noteworthy that at the time of the first interviews there was still a concern that the hospital system would imminently be overwhelmed with COVID-19 patients requiring intensive care. Hence the AiMs obligations to the team were contextualised in the moment of crisis. However as the ability of the AiM to contribute grew throughout the time of the AiM, their value to the team was apparent in the second interviews despite the moment of crisis having passed. AiMs continue to be employed in NSW Health facilities in 2022.

### Implications/recommendations

For final year students, the expectation to contribute to the care of patients should be formalized. Having a position description which outlined skills and responsibilities assigned to their role enabled AiMs to work effectively [23]. Whilst payment was a factor in cementing this contract, AiMs identified obligations to team members and the care of patients that relied on social reciprocity rather than monetary reward. Final year medical students could benefit from a position description that identifies them as a member of a patient care team. Similar to assistantships in the UK [12], explicit obligations in the patient care team are fundamental to the quality of the experience. Small token financial renumeration may motivate students and team members to commit time to establishing relationships for system benefits. Placing students within a specialty in line with their career aspirations may be an alternative incentive towards active participation in final year. Future AiMs are likely to benefit from an increased understanding of the role. A culture of service to the health system can be developed whereby final year students can experience authentic learning and contribute to care. Access to the eMR as recommended by NSW Health [23] would recognize AiMs’ contribution, enable it to be useful within the team, and allow authentic learning.

### Limitations

Our study was based on students from one medical school, but conducted across very different urban and rural health service settings. Findings were consistent across these diverse settings and strengthens this analysis. The interviewers may have been known to AiMs, which could have influenced their responses, although the large number of interviewers would have diluted any effect. There was consistency in data collection with shared interview protocols being used for both individual and group interviews across sites. Students that were offered AiMS were higher performing and hence may have been more able to be trusted to be involved in patient care. Extending the program to all final year medical students may not result in the same positive findings for student learning.

The application of Activity theory is not new to medical education research and is particularly useful in experiential learning environments where there is often contested objectives [2]. Activity theory has several generations of theory [27] with the third generation considering interactions between multiple activity systems. This research has utilized second generation Activity theory, which is focused on the workplace. University tools, rules and requirements potentially impacted on AiMs and could be considered utilizing third generation Activity theory. This was an interview study, without the direct observations of ethnographic studies, however multiple rounds of discussion among team members with knowledge of the AiM environment contributed to understanding the data in context. Utilizing an Activity theory lens in the development of future research would further understanding of medical students’ experiential learning and may contribute to Activity theory’s ongoing refinement.

Whilst COVID-19 has disrupted medical education and forced many programs to online delivery [33] the AiM program brought medical students more fully into the clinical space. This paper has explored AiMs perspectives of the role and through the lens of Activity theory demonstrated opportunities to enhance the final medical student year.

## Conclusion

The AiM role and particularly its implementation within the context of the COVID-19 pandemic was clearly unique and mobilised many people to achieve success. As a result, a truly new position was developed, neither medical student nor doctor.

Medical students were successfully included in medical teams as AiMs. However, consideration of an ongoing AiM type program for Australian final year medical students is not solely a response to COVID-19. The role provides a useful intermediate step in the transition to PGY1 allowing students to learn and contribute meaningfully to medical teams. Ensuring teams are structured with space for a dedicated assistant role and access to the eMR enable a successful assistant experience and should also be considerations for any final year medical student placements.

## Supplementary Information

**Additional file 1.**

**Additional file 2.**

## Data Availability

The datasets generated and analysed during the current study are not publicly available due to the consent obtained but are available from the corresponding author on reasonable request.

## References

[1] Fenwick T, Zukas M, Kilminster S, Bullock A, Fox F, Barnes R (2013). Transitions in medicine: trainee doctor stress and support mechanisms. J Work Learn.

[2] Gillespie H, McCrystal E, Reid H, Conn R, Kennedy N, Dornan T (2021). The pen is mightier than the sword. Reinstating patient care as the object of prescribing education. Med Teach.

[3] de Lasson L, Just E, Stegeager N, Malling B (2016). Professional identity formation in the transition from medical school to working life: a qualitative study of group-coaching courses for junior doctors. BMC Med Educ.

[4] Australian Health Ministers Advisory Council. Review of Medical Intern Training Discussion Paper. Australia; 2015. Available from http://www.coaghealthcouncil.gov.au/medicalinternreview.

[5] Sturman N, Tan Z, Turner J (2017). “A steep learning curve”: junior doctor perspectives on the transition from medical student to the health-care workplace. BMC Med Educ.

[6] Levy K, Voit J, Gupta A, Petrilli CM, Chopra V (2016). Examining the July Effect: a national survey of academic leaders in medicine. Am J Med.

[7] Burford B, Whittle V, GHS V. The relationship between medical student learning opportunities and preparedness for practice: a questio nnaire study. BMC Med Educ. 2014;14(223). 10.1186/1472-6920-14-223. 10.1186/1472-6920-14-223 PMC4288662 25331443

[8] Weaver R, Peters K, Koch J, Wilson I (2011). 'Part of the team': professional identity and social exclusivity in medical students. Med Educ.

[9] Violato CCM, Englander R (2021). Validity Evidence for Assessing Entrustable Professional Activities During Undergraduate Medical Education. Acad Med.

[10] Chaou CH, Yu SR, Chang YC, Ma SD, Tseng HM, Hsieh MJ, et al. The evolution of medical students' preparedness for clinical practi ce during the transition of graduation: a longitudinal study from the undergraduate to postgraduate periods. BMC Med Educ. 2021;21(1). 10.1186/s12909-021-02679-8. 10.1186/s12909-021-02679-8 PMC8101179 33957907

[11] Yardley S, Teunissen P, Dornan T. Experiential learning: AMEE guide No. 63. Medical Teacher. 2012;34:e102–15. 10.3109/0142159X.2012.650741. 10.3109/0142159X.2012.650741 22289008

[12] Crossley JG, Vivekananda-Schmidt P (2015). Student assistantships: bridging the gap between student and doctor. Adv Med Educ Pract.

[13] van den Broek S, Querido S, Wijnen-Meijer M, van Dijk M, ten Cate O (2020). Social Identification with the Medical Profession in the Transition from Student to Practitioner. Teach Learn Med.

[14] Worley P, Couper I, Strasser R, Graves L, Cummings BA, Woodman R (2016). A typology of longitudinal integrated clerkships. Med Educ.

[15] Looi JC, Yong CS (2017). The doctor as an expert: apprentice, journeyman or master. Med J Aust.

[16] Rose S (2020). Medical Student Education in the Time of COVID-19. JAMA..

[17] Australian Medical Student Association (AMSA). Coordinated Response Required for Medical Student Involvement in COVID-19. Kingston, ACT: Australian Medical Student Association (AMSA). https://www.amsa.org.au/blog/coordinated-response-required-medical-student-involvement-covid-19; 2020. p. 2.

[18] Mahase E (2020). Covid-19: medical students to be employed by NHS. BMJ.

[19] Medical Deans Australia and New Zealand. Medical Students’ contribution to the health workforce response to COVID-19 2020 [Available from: https://medicaldeans.org.au/md/2020/03/2020-March-19_medical-students-contribution-to-the-COVID-19-health-workforce.pdf.

[20] Tong A, Sainsbury P, Craig J (2007). Consolidated criteria for reporting qualitative research (COREQ): a 32-item checklist for interviews and focus groups. Int J Qual Health Care.

[21] Health Education and Training Institute NSW. The Intern Guide; A resource for junior doctors, their educators and supervisors. 2016. Available from https://www.heti.nsw.gov.au/education-and-training/courses-and-programs/prevocational-education/the-intern-guide.

[22] Western Sydney University. Unit Outline; School of Medicine; 400978 - Integrated Clinical Rotations 4. 2021. Accessed from https://hbook.westernsydney.edu.au/subject-details/medi4003/.

[23] NSW Ministry of Health. NSW Health Assistant in Medicine Evaluation Report. 2021. Report No.: 978–1–76081–739–8. Available from https://www.health.nsw.gov.au/workforce/medical/Pages/aim-evaluation-report.aspx.

[24] Monrouxe LV, Hockey P, Khanna P, Klinner C, Mogensen L, Mara DA (2021). Senior medical students as assistants in medicine in COVID-19 crisis: a realist evaluation protocol. BMJ Open.

[25] Braun VCV (2006). Using thematic analysis in psychology. Qual Res Psychol.

[26] Sannino A, Engeström Y, Lemos M (2016). Formative Interventions for Expansive Learning and Transformative Agency. J Learn Sci.

[27] Engestrom Y (2015). Learning by Expanding: An Activity-Theoretical Approach to Developmental Research.

[28] Klitgaard TL, Stentoft D, Skipper M, Gronkjaer M, Nohr SB (2021). Struggling to fit the white coat and the role of contextual factors within a hospital organisation - an ethnographic study on the first months as newly graduated doctors. BMC Med Educ.

[29] Engestrom Y, Pyorala E (2021). Using activity theory to transform medical work and learning. Med Teach.

[30] QSR International Pty Ltd. NVivo (Version 12). 2018. Available from https://www.qsrinternational.com/nvivo-qualitative-data-analysis-software/home.

[31] Doris Ø, Lundsgaard K, Tolsgaard M, Mortensen O, Mylopoulos M, Østergaard D (2019). Title: Embracing Multiple Stakeholder Perspective s in Defining Trainee Competence Embracing Multiple Stakeholder Perspectives in Defining Trainee Competence. Acad Med..

[32] Hirsh DA, Holmboe ES, ten Cate O (2014). Time to Trust: Longitudinal Integrated Clerkships and Entrustable Professional Activities. Acad Med..

[33] Daniel M, Gordon M, Patricio M, Hider A, Pawlik C, Bhagdev R (2021). An update on developments in medical education in response to the COVID-19 pandemic: A BEME scoping review: BEME Guide No. 64. University of Toronto. Med J.

