# Correction: “You’re actually part of the team”: a qualitative study of a novel transitional role from medical student to doctor

**DOI:** 10.1186/s12909-023-04127-1

**Published:** 2023-03-15

**Authors:** Natalie Edmiston, Wendy Hu, Stephen Tobin, Jannine Bailey, Caroline Joyce, Krista Reed, Lise Mogensen

**Affiliations:** 1 School of Medicine, Western Sydney University, Sydney, Australia; 2 University Centre for Rural Health, Lismore, Australia

**Correction: BMC Med Educ 23, 112 (2023)**

**https://doi.org/10.1186/s12909-023-04084-9**

Following publication of the original article [1], we have been informed that first name and last name have been swapped for all the authors.

The author group has been updated above and the original article has been corrected.

## References

[1] Edmiston (2023). “You’re actually part of the team”: a qualitative study of a novel transitional role from medical student to doctor. BMC Med Educ.

