# Supplementary material for: “You’re actually part of the team”: a qualitative study of a novel transitional role from medical student to doctor
Source: BMC Med Educ. 2023 Feb 15;23:112. doi: 10.1186/s12909-023-04084-9 (PMC9930018; doi:10.1186/s12909-023-04084-9)
Supplement: Supplementary file 1 — Additional file 1. [file 12909_2023_4084_MOESM1_ESM.docx]

APPENDIX A

**ROUND ONE INTERVIEW QUESTIONS**

**BECOMING AND BEING AN ASSISTANT IN MEDICINE**

**PREAMBLE**

[Introduce self and relationship – nil/minimal – to the AiM program and assessment]

Thank you for taking the time to join this discussion group about your experiences to date as an AiM.

You should all have received a link to the ‘Building Evidence in Medical Education’ project information and consent form. If not here it is again (link in chat ) please take a moment to read the information and complete the consent form. You will see that there is a question at the end about contacting you for a second focus group when or soon after your AiM role concludes. Saying yes, will only allow us to email you an invitation, it doesn’t bind you to participate again.

From what we’ve heard, everyone has different roles, so we are very interested to hear what it’s been like for you. There are definitely no right or wrong answers. We’d like you to be honest about the lows, as well as the highs. Anything you say will be kept confidential by us, and all data will be de-identified before analysis. We ask that you also respect the privacy of others in this group.

To assist the flow of discussion we would like to record it. Afterwards, the recording will be reviewed and all responses de-identified for analysis.

[If using Zoom. We’d also like you to use the Chat function so that everyone has a chance to express their views. We will also use anonymised Chat as data]

At the end of the session we’d like you to complete a 5 min (online) questionnaire about your background. Personal information will also be kept confidential and separate from the discussions, but will help with context for analysing the data. (For Zoom interviews, email link and allocate time to complete).

If for any reason you would like to leave the session, you are free to do so. If something is raised that causes distress, please let me know [private chat] and we will follow up with you afterwards to make sure you are supported.

Today we have four topics that we would like to hear your thoughts and experiences on. We’d like to hear what it’s been like for everyone, from before the start of the AiM role, starting the role and till now. Where we can, we will use what you say today to improve the rest of your AiM time.

Do you have any questions before we start?

Is it OK to start recording now?

[On Zoom make sure transcription is on and record to the cloud]

**OPENING**

To start with….

**SECTION 1: BEFORE BECOMING AN AiM**

- **Tell me why you decided to apply for the Assistant in Medicine role?**
  - To you, what was the purpose of the AiM role?
  - What did you expect from being an AiM?
  - Probe any generic phrases from motivation – You suggested (e.g. service to community, opportunity to learn etc) – can you explain?
  - What did you expect to do/learn from this?
  - What were your concerns, if any, about the AiM role?

**SECTION 2: STARTING AS AN AiM**

- **Tell me about the process of becoming an AiM**
  - What was the employment process like?
  - Did anything about the process surprise you?
  - Was anything about the process difficult?
  - What orientation did you receive at the hospital/health service?
  - Who ran the orientation? How well did it prepare you for what came next?
  - From where you are now, what (knowledge, skills) should it have covered?
- **Thinking back to those first week(s), tell me what your day was like as an AiM.**
  - Work hours? Who did you meet or work with first? Who else is on your team? When did you meet them?
  - What surprised or challenged you in those first days? What happened? How did you respond?
  - What tasks were you given to do?
  - What were your feelings like in those first days?
  - How do you feel now, looking back on those days?

**SECTION 3: BEING AN AiM**

- **Compared to being a 5^th^ year student, what is different about being an AiM?**
  - In what ways have you had to “step up”?
  - What new responsibilities have you adopted/ undertaken?
  - Do you feel you are a student, or an intern, or something else? [Probe what this means in how they act, think, talk and feel?]
  - Has the role changed since you started? In what way?
- **How have you been/are you being supported in your role?**
  - Who are your main supports? E.g. Supervisors, team members, others on ward/service/hospital departments e.g. DPET, JMO Manager, DAIMs, peers, university academics and professional staff.
  - What do each of these do to support you?
- **What feedback have you received, or sought, about how you are doing?**
  - From whom have you received supervision, guidance and/or feedback about your role? How has this affected your role?
  - In what form has your feedback been given? E.g verbal informal, mid-term appraisals, EPAs….
- **How have you found the education sessions?**
  - Relevance, best/least useful, ideas for improvement, future sessions, usefulness of recorded sessions.
  - What other training or education have you received/undertaken while being an AiM?
  - In what ways have the School’s training/communication sessions/other activities, helped prepare for the AiM role? In what ways have they not? What could be different?

**SECTION 4: LOOKING FORWARDS, AS AN AiM**

- **What would you like to be different, compared to these first weeks?**
  - What could the health service/team/supervisor do differently?
  - What could the university/clinical school do differently?
  - What training opportunities relevant to your role would be useful?
- **Is there anything else anyone would like to add?**

**CLOSING**

Thank you for all your responses so far.

Thank you for your time today. We will be reviewing your responses along with those from the other groups, to see what could be improved for the rest of the AiM time and to improve future AiM roles.

We hope that talking about your experiences has helped you to reflect on how far you have come. These reflections may be useful for your portfolio.

If you would like more information about this evaluation, or have concerns or follow up comments please contact xxxx

If you have any issues or concerns about your AiM role or anything related to your role, please contact xxxx
