# Supplementary material for: “You’re actually part of the team”: a qualitative study of a novel transitional role from medical student to doctor
Source: BMC Med Educ. 2023 Feb 15;23:112. doi: 10.1186/s12909-023-04084-9 (PMC9930018; doi:10.1186/s12909-023-04084-9)
Supplement: Supplementary file 2 — Additional file 2. [file 12909_2023_4084_MOESM2_ESM.docx]

APPENDIX B

**ROUND TWO INTERVIEW QUESTIONS**

**BECOMING AND BEING AN ASSISTANT IN MEDICINE**

**PREAMBLE**

[Introduce self and relationship – nil/minimal – to the AiM program and assessment]

Thank you for taking the time [again] to join this discussion group/talk about your experiences as an AiM.

You should all have received a link to the ‘Building Evidence in Medical Education’ project information and consent form. If not here it is again (link in chat) please take a moment to read the information and complete the consent form.

From what we’ve heard in the first round, everyone has had different experiences, so we are very interested to hear from you. There are definitely no right or wrong answers. We’d like you to be honest about the lows, as well as the highs. Anything you say will be kept confidential by us, and all data will be de-identified before analysis. We ask that you also respect the privacy of others in this group.

To assist the flow of discussion we would like to record it. Afterwards, the recording will be reviewed and all responses de-identified for analysis.

[If using Zoom. We’d also like you to use the Chat function so that everyone has a chance to express their views. We will also use anonymised Chat as data]

If for any reason you would like to leave the session, you are free to do so. If something is raised that causes distress, please let me know [private chat] and we will follow up with you afterwards to make sure you are supported.

Today we would like to build on some of the questions we asked last time to find out how you, the role and the context your work in might have changed or stayed the same. Where we can, we will use what you say today to improve the AiM role for the future.

**Do you have any questions before we start? Is it OK to start recording now?**

**OPENING**

**SECTION 1: Being in the AiM role - Following up from last interview**

- **Tell me what you have been doing in your Assistant in Medicine role since we last spoke?**
- Has anything changed about your role? (New role, new team, new team members?)
- What have been your main tasks and responsibilities?
- What new responsibilities have you adopted/ undertaken? Were these the tasks and responsibilities you expected in the role?
- What else did you expect from being an AiM?
- What surprises or highlight, concerns or challenges have you met since we last spoke? What happened? How did you respond?

**Section 2: Professional Development and Identity**

- **Now that you have been in the AiM role for many weeks has anything changed for you?**
  - Has anything changed about the way you work, the way you feel about the role or the way you think of yourself in the role?
  - Has anything changed about the way you go about your work, or
  - The way others respond to you or treat you in the role?
- **Do you feel that you have changed from working in this role?**
  - What new responsibilities have you adopted/ undertaken? Were these the tasks and responsibilities you expected in the role?
  - Probe if appropriate: What is it about the AiM role that may make you more likely to take on new responsibilities? Is this different from being a 5th year student?
- **At this point do you feel like a medical student or trainee doctor – or something in-between?**
  - How? What has made you feel this way?
  - What this means in how they act, think, talk and feel?
  - How have you managed the changing responsibilities between being a student and being staff?
  - How do you feel about becoming a full time student again?

**SECTION 3: Learning opportunities and experiences in the AiM role**

- **How did you experience and learn from working in a medical team?**
  - What helped or hindered you in getting comfortable in the role/ get on with the job? In what way? Was this similar or different in the different teams?
  - How do you see yourself in the team? Do you feel like you or your role has become integral to the team?
- **What other opportunities for learning have you had/taken while being in this role?**
  - How have these helped you in your role? Knowledge? Skills?
  - Are these opportunities different from what has been available to you as a student?
- **What opportunities did you have to work with colleagues from other health professions?**
  - Probes if relevant: How did you find a multidisciplinary approach changed the care of patients? Can you give an example?
  - How did you find this experience change your understanding of the clinical presentation or management options? If any, what key learning experiences from being part of a multi-disciplinary team do you think you will implement in your own practice in the future?
- **How have you been/are you being supported in your (new) role?**
  - Has there been a change in the way you access/receive support during your time in the role?
  - Who are your main supports? (E.g. Supervisors, team members, others on ward/service/hospital departments e.g. DPET, JMO Manager, DAIMs, peers, university academics and professional staff).
  - What do each of these do to support you?
- **What feedback have you received, or sought, about how you are doing?**
  - [Focus on change from last team if repeat or since starting if new participant].
  - Who has been giving feedback? When? How often?
  - What has been the most useful form of feedback?
  - **What has been your experiences in using the EPAs?**
  - How easy/ difficult are they to use while on the job? What did you learn from doing them (if anything?)

**SECTION 4: LOOKING BACK – LOOKING FORWARD**

**Reflecting on your experiences as an AiM**

- **Overall, how well has the AiM role met your expectations?**
  - What has matched your expectations? What has not?
  - What have your gained or missed by taking on this role?
- **How do you think being an AiM prepares/ does not prepare for internship?**
  - Do you feel prepared for starting internship? What experiences in particular have prepared you?
  - If anything, what would help you feel better prepared?
- **If the AiM role were to be retained next year, what aspects would you keep, what would you change?**
  - How could the AiM experience be improved? Why? How?
- **Is there anything else anyone would like to add?**

**CLOSING**

Thank you for all your responses and your time today. We will be reviewing your responses to see what could be improved for future AiM roles.

We hope that talking about your experiences has helped you to reflect on how far you have come.

If you would like more information about this evaluation, or have concerns or follow up comments please contact xxxx

If you have any issues or concerns about your AiM role or anything related to your role, please contact xxxx
